# Transition between modalities of renal replacement therapy

**DOI:** 10.1590/2175-8239-JBN-2024-E008en

**Published:** 2024-09-06

**Authors:** Thyago Proença de Moraes, Caio Pellizzari

**Affiliations:** 1 Pontifícia Universidade Católica do Paraná, Curitiba, PR, Brazil.

The transition between renal replacement therapy modalities is common and often poses major challenges for both the nephrology team and the patients and their families^1,2,3^. If it is not conducted in a planned manner and following a pre-established course, in an attempt to recognize and overcome the traditional barriers faced at this moment, the outcomes may be negative, as is often the case^4^. For these reasons, an initiative called INTEGRATED recently convened a group of experts on the subject to design a guideline for peritoneal dialysis that would optimize these outcomes^5^. A great deal of the topics addressed in this document can be observed in the study recently published in the Brazilian Journal of Nephrology by the Portuguese group of Francisco et al.^6^

Several factors presented by patients transitioning to PD from another RRT are described as potential barriers. Patients migrating from hemodialysis may be divided into two groups: those who required urgent therapy, and those who opted for HD as their first modality and are now transitioning to PD due to vascular access failure or hemodynamic instability. This differentiation is important, as the second group generally consists of patients who most likely did not want to switch therapies and often face difficulties when assuming responsibility for home treatment. Additionally, these patients generally have a longer history of kidney disease and often start PD with a lower urine output, or even anuria. This was not the case in the study by Francisco et al, which demonstrated that the patients’ residual diuresis was similar to that of patients who started on PD as their first choice^6^. Conversely, the transition of transplant patients seems to have occurred at a much later stage, since at the beginning of PD follow-up, the GFR of this subgroup was on average 3 times lower than that of the other 2 subgroups.

Patients who start on PD with lower GFR usually require a much greater glucose exposure to achieve adequate ultrafiltration. This exposure is known to interfere with the health of the peritoneal membrane, and may even be associated with a higher risk of all-cause mortality^7^. Thus, it was understandable to note that the study by Francisco et al. revealed a higher rate of UF failure among patients transitioning from transplantation.

Finally, when designing the study, the authors believed in the hypothesis that patients who switch from a previous RRT modality to PD are at increased risk of negative outcomes. The hypothesis was not confirmed, and similar outcomes were observed between the study groups regarding mortality and transfer to HD. However, this finding may be related to several factors present in this study, including its retrospective nature, being a single-center study, and, most notably, the small number of patients in the HD and transplant subgroup, even with the long follow-up period of the cohort. Despite the limitations, and as the authors correctly mention, there is no doubt that PD should be an option for patients from other renal replacement therapies. Our major problem at present is precisely to improve understanding on how to deal with the transition process, including some easily identifiable factors (Chart 1), but often difficult to manage. It is important to consider that the transition process from PD to HD is even more common than the reverse and is also very poorly explored in literature.

**Chart 1:** Key factors in the transition process

Duration between the decision to switch and the actual transition	Does a sudden transition have an impact on outcomes? Would informing the patient about a potential transition right from the beginning of the current therapy change their acceptance? What is the ideal time to proceed with the transition?
Change in patient care team	What is the best way to execute a transition without compromising the information flow and interaction between the new team and the previous one? How can the multidisciplinary team be trained for a more effective transition? How to monitor patients during the transition?
Patient preparation for the transition process	Are we properly providing information on the different RRT options (without bias)? How to communicate the need for transition when the patient has no other options? How to involve the patient’s family in the process? What resources can we provide to patients and their families to better deal with psychological aspects?
Influence of clinical factors	How to monitor and manage clinical determinants that impact the transition? Are there “warning signs” in current care protocols to indicate those at a higher risk of short-term transition?
