# Supplementary material for: Transition between modalities of renal replacement therapy
Source: J Bras Nefrol. 2024 Sep 6;46(3):e2024E008. doi: 10.1590/2175-8239-JBN-2024-E008en (PMC11379404; doi:10.1590/2175-8239-JBN-2024-E008en)
Supplement: Translation

# Translation: Transição entre modalidades de terapia renal substitutiva

**DOI:** 10.1590/2175-8239-JBN-2024-E008pt

**Authors:** Thyago Proença de Moraes, Caio Pellizzari

**Affiliations:** 1 Pontifícia Universidade Católica do Paraná, Curitiba, PR, Brasil.

A transição entre modalidades de terapia renal substitutiva é comum e frequentemente impõe importantes desafios tanto para a equipe da nefrologia quando para o paciente e sua família^1,2,3^. Se não for realizada de maneira planejada e seguindo um fluxo pré-estabelecido, na tentativa de reconhecer e superar as tradicionais barreiras presentes nesse momento, os desfechos podem se mostrar negativos, como frequentemente acontece^4^. Por esses motivos, uma iniciativa chamada INTEGRATED recentemente reuniu experts no assunto para a elaboração de uma diretriz dentro da diálise peritoneal que otimizasse esses desfechos^5^. Grande parte dos temas abordados nesse documento pode ser observada no trabalho há pouco publicado no *Brazilian Journal of Nephrology* pelo grupo português de Francisco et al.^6^

Diversos fatores apresentados por pacientes que migram para a DP de outra TRS são descritos como potenciais barreiras. Pacientes que migram da hemodiálise podem ser divididos em dois grupos, aqueles que precisaram de terapia com urgência e aqueles que optaram pela HD como primeira modalidade e estão migrando para a DP por questões de falha de acesso vascular ou por instabilidade hemodinâmica. Essa diferenciação é importante, visto que o segundo grupo geralmente é composto por pacientes que provavelmente não desejavam mudar de terapia e costumam enfrentar dificuldades ao assumir a responsabilidade de fazer o tratamento em casa. Além disso, são pacientes em geral com mais tempo de doença renal, e frequentemente chegam à DP com um volume de diurese menor, ou mesmo anúricos. Esse não foi o caso do estudo de Francisco e cols., em que foi demonstrado que a diurese residual dos pacientes era semelhante à dos pacientes que iniciaram na DP como primeira escolha^6^. Por outro lado, a transição do paciente oriundo do transplante parece ter ocorrido em um momento muito mais tardio, já que no início do seguimento da DP a TFG desse subgrupo era em média 3 vezes menor que nos outros 2 subgrupos.

Pacientes que iniciam em DP com menor TFG habitualmente necessitam de uma exposição à glicose muito maior para se obter ultrafiltração adequada, e essa exposição sabidamente interfere na saúde da membrana peritoneal, podendo inclusive se associar a um risco mais elevado de mortalidade por todas as causas^7^. Desse modo, foi compreensível observar que o estudo de Francisco & cols. demonstrou uma taxa mais elevada de falência de UF entre os pacientes que migraram do transplante.

Finalmente, quando projetaram o estudo, os autores acreditavam na hipótese de que pacientes que passam de uma modalidade prévia de TRS para a DP têm maior risco de um desfecho negativo. A hipótese não foi confirmada, e desfechos semelhantes foram observados entre os grupos estudados quanto à mortalidade e transferência para HD. Entretanto, esse achado pode estar relacionado a diversos fatores presentes nesse estudo, incluindo seu caráter retrospectivo, ser de um único centro e, principalmente, pelo pequeno número de pacientes no subgrupo oriundo da HD e do transplante, mesmo com o longo tempo de seguimento da coorte. Apesar das limitações, e como mencionam corretamente os autores, não há dúvida de que a DP deva ser uma opção para pacientes oriundos de outras terapias renais substitutivas. Nosso maior problema atualmente é justamente aumentar a compreensão de como lidar com o processo de transição, incluindo alguns fatores facilmente identificáveis (Quadro 1), mas frequentemente difíceis de ser manejados, sempre levando em conta que o processo de transição da DP para HD é ainda mais comum que o inverso e também muito pouco explorado na literatura.

**Quadro 1:** Fatores importantes no processo de transição

Duração entre a decisão da mudança e a efetiva transição	Uma mudança abrupta impacta os desfechos? Informar o paciente sobre possível transição desde o início da terapia atual mudaria sua aceitação? Qual o tempo ideal para se proceder a transição?
Mudança na equipe de assistência ao paciente	Qual a melhor maneira de executar uma mudança sem comprometer o fluxo de informações e a interação entre a nova equipe e a anterior? Como capacitar a equipe multiprofissional para uma transição mais efetiva? Como fazer o monitoramento dos pacientes durante a transição?
Preparação do paciente para o processo de mudança	Estamos informando corretamente sobre as diferentes TRS (sem viés)? Como deve ser dada a notícia da necessidade de transição quando o paciente não tem opção? Como envolver a família do paciente no processo? Quais recursos podemos fornecer ao paciente e aos familiares para melhor lidar com aspectos psicológicos?
Influência de fatores clínicos	Como acompanhar e manejar determinantes clínicos que impactam a transição? Nos protocolos de cuidado atuais há “sinais de alerta” para mostrar quem tem maior risco de transição em curto prazo?
